# Dynamic changes in the subcellular distribution of the tobacco ROS-producing enzyme RBOHD in response to the oomycete elicitor cryptogein

**DOI:** 10.1093/jxb/eru265

**Published:** 2014-07-01

**Authors:** Elodie Noirot, Christophe Der, Jeannine Lherminier, Franck Robert, Pavla Moricova, Kiên Kiêu, Nathalie Leborgne-Castel, Françoise Simon-Plas, Karim Bouhidel

**Affiliations:** ^1^INRA, UMR1347 Agroécologie, ERL CNRS 6300, Plateforme DImaCell, Centre de Microscopie INRA/Université de Bourgogne, BP 86510, F-21065 Dijon Cedex, France; ^2^Université de Bourgogne, UMR1347 Agroécologie, ERL CNRS 6300, BP 86510, F-21065 Dijon Cedex, France; ^3^INRA, UMR1347 Agroécologie, ERL CNRS 6300, BP 86510, F-21065 Dijon Cedex, France; ^4^ Present address: Department of Biochemistry, Faculty of Science, Palacký University in Olomouc, Šlechtitelů 11, CZ-783 71 Olomouc, Czech Republic; ^5^INRA, UR341 Mathématiques et Informatique Appliquées, F-78352 Jouy-en-Josas Cedex, France

**Keywords:** BY-2 cells, cryptogein, *Nicotiana tabacum*, protein trafficking, respiratory burst oxidase homolog D (RBOHD), reactive oxygen species, protein trafficking.

## Abstract

Plant NADPH oxidases, also known as respiratory burst oxidase homologues (RBOHs), have been identified as a major source of reactive oxygen species (ROS) during plant–microbe interactions. The subcellular localization of the tobacco (*Nicotiana tabacum*) ROS-producing enzyme RBOHD was examined in Bright Yellow-2 cells before and after elicitation with the oomycete protein cryptogein using electron and confocal microscopy. The plasma membrane (PM) localization of RBOHD was confirmed and immuno-electron microscopy on purified PM vesicles revealed its distribution in clusters. The presence of the protein fused to GFP was also seen in intracellular compartments, mainly Golgi cisternae. Cryptogein induced, within 1h, a 1.5-fold increase in RBOHD abundance at the PM and a concomitant decrease in the internal compartments. Use of cycloheximide revealed that most of the proteins targeted to the PM upon elicitation were not newly synthesized but may originate from the Golgi pool. ROS accumulation preceded *RBOHD* transcript- and protein-upregulation, indicating that ROS resulted from the activation of a PM-resident pool of enzymes, and that enzymes newly addressed to the PM were inactive. Taken together, the results indicate that control of RBOH abundance and subcellular localization may play a fundamental role in the mechanism of ROS production.

## Highlight text

The oomycete elicitor cryptogein triggers the relocation of RBOHD from intracellular compartments to the plasma membrane in tobacco cells. This suggests that intracellular trafficking is a potential determinant of RBOHD activity.

## Introduction

Reactive oxygen species (ROS) such as hydrogen peroxide (H_2_O_2_), superoxide anions (O_2_^•–^), hydroxyl radicals (OH^•^), and singlet oxygen (^1^O_2_) are natural by-products of plant cell metabolism that must be detoxified to protect cellular functions from their strong oxidative properties (Halliwell, 2006). A transient increase in ROS production is observed when a plant is exposed to a pathogen, and is referred to as the oxidative burst. ROS produced during the oxidative burst are thought to be involved in defence reactions in several ways: directly as antimicrobial agents (Chen and Schopfer, 1999) or cross-linking agents during cell wall reinforcement (Bradley *et al.*, 1992), and indirectly as signalling molecules to trigger the hypersensitive response (HR) and systemic acquired resistance (SAR) (Torres, 2010). The oxidative burst is usually biphasic with a rapid and transient phase within 1h of pathogen recognition followed by a long-lasting second phase a few hours later that is thought to be responsible for the HR (Lamb and Dixon, 1997). Primary ROS production is predominantly apoplastic and, depending on the plant species, is dependent upon plasma membrane (PM) NADPH oxidase, cell wall peroxidase, or both (O’Brien *et al.*, 2012).

Plant NADPH oxidases, also designated as respiratory burst oxidase homologues (RBOHs), belong to a small family of highly conserved proteins with ten members in the model plant *Arabidopsis thaliana* (Torres *et al.*, 1998; Marino *et al.*, 2011). RBOHs are membrane proteins composed of six transmembrane domains associated with two haem groups, a C-terminal region with NADPH- and FAD-binding domains, and an N-terminal regulatory region with two calcium-binding EF-hands and phosphorylation domains (Suzuki *et al.*, 2011). RBOHs catalyse the formation of the superoxide anion O_2_^•–^ by transferring an electron from intracellular NADPH to an apoplastic molecule of oxygen (O_2_) (Sagi and Fluhr, 2006). Superoxide anions, which are short-lived radicals, are then rapidly converted to hydrogen peroxide either spontaneously or by superoxide dismutase (Mori and Schroeder, 2004).

The role of RBOHs in plant–pathogen interactions has been investigated in different plant species using knockout mutants and antisense approaches. In *Arabidopsis*, the RBOHD and RBOHF isoforms are responsible for the ROS burst in response to MAMPs (microbe–associated molecular patterns) (Zhang *et al.*, 2007), DAMPs (damage-associated molecular patterns) (Galletti *et al.*, 2008), and microbial pathogens (Torres *et al.*, 2002; Pogany *et al.*, 2009), but with a prevalent role for RBOHD. In *Nicotiana benthamiana* the orthologues of AtRBOHD and AtRBOHF, named RBOHB and RBOHA, respectively, are the principal ROS producers in response to infection by *Phytophthora infestans* (Yoshioka *et al.*, 2003) or *Botrytis cinerea* (Asai and Yoshioka, 2009), and to elicitors of plant defence reactions (Asai *et al.*, 2008; Zhang *et al.*, 2009). In tobacco (*Nicotiana tabacum*), RBOHD is the sole isoform responsible for the ROS burst in response to cryptogein (Simon-Plas *et al.*, 2002; Lherminier *et al.*, 2009), a proteinaceous elicitor secreted by the oomycete *Phytophthora cryptogea* known to induce the HR and SAR (Ricci *et al.*, 1989; Keller *et al.*, 1996).

Opposite results were obtained regarding the effects of the RBOH-mediated ROS burst on the HR and resistance to pathogens. In *Arabidopsis*, the *rbohD*/*rbohF* double mutant displayed a reduced HR and unaffected pathogen growth after inoculation with an avirulent *Pseudomonas syringae* strain, but an enhanced HR and resistance to the biotrophic oomycete *Peronospora parasitica* (Torres *et al.*, 2002). In *N. benthamiana*, the *rbohB*/*rbohA* double mutant exhibited reduced HR and enhanced susceptibility after infection with the avirulent oomycete *Phytophthora infestans* (Yoshioka *et al.*, 2003). These diverse effects on the HR and disease resistance suggest that RBOH-derived ROS are not simple toxic compounds triggering cell death, but components of signalling pathways that may have opposite effects on plant defence reactions (Torres *et al.*, 2005; Torres, 2010; Marino *et al.*, 2011). A long-distance signalling function has been demonstrated for RBOHD-derived ROS in *Arabidopsis* in response to various abiotic stresses (Miller *et al.*, 2009). Signalling activities of RBOH-derived ROS are probably also modulated by other ROS sources (Bindschedler *et al.*, 2006; Yoda *et al.*, 2006; Daudi *et al.*, 2012) and other signalling molecules. One of them is nitric oxide (NO) that is synthesized following pathogen recognition within the same time frame (Romero-Puertas *et al.*, 2004). Both NO and ROS are known to react with each other to produce pro-death molecules such as singlet oxygen or hydroxyl radicals, and a balanced production between intracellular ROS and NO has been shown to be a key determinant for the HR (Delledonne *et al.*, 2001). The recent finding that NO-mediated *S*-nitrosylation of AtRBOHD governs a negative feedback loop limiting the production of ROS and the HR (Yun *et al.*, 2011) has shed some light on the mostly unknown molecular mechanisms that underpin the interplay between NO and ROS.

To get a better understanding of the cellular and physiological functions of RBOH-derived ROS as ephemeral signal molecules in plant–microbe interactions, it is essential to determine the subcellular localization and dynamics of their producers. Cell fractionation and immunolocalization studies were the first to reveal that RBOHs are intrinsic PM proteins of plant cells (Keller *et al.*, 1998; Sagi and Fluhr, 2001; Simon-Plas *et al.*, 2002), a result later confirmed with the use of GFP-fusions (Kobayashi *et al.*, 2006; Takeda *et al.*, 2008). Proteomic studies also showed that RBOHs are present in detergent-insoluble fractions (DIMs) of the PM thus suggesting that, like their animal counterparts, they could be associated *in vivo* with sterol- and sphingolipid-enriched domains also known as membrane rafts (Mongrand *et al.*, 2004; Morel *et al.*, 2006; Fujiwara *et al.*, 2009). The non-uniform distribution of several RBOHs within the PM of differentiating cells is in line with this finding (Takeda *et al.*, 2008; Liu *et al.*, 2009; Lee *et al.*, 2013). However, the cell fate of RBOHs after pathogen perception is not documented.

The focus of the present study was to characterize the subcellular localization of RBOHD in tobacco Bright Yellow-2 (BY-2) cells before and after elicitation with cryptogein using confocal and electron microscopy. Two RBOHD isoforms were identified in BY-2 cells. The RBOHDs were found to reside in the PM in the form of small clusters in the Golgi cisternae and in a second, as yet unidentified, intracellular compartment. The RBOHDs partitioning between the PM and endomembranes were further shown to be altered following elicitation. Finally, examination of the relationships between transcriptional control, subcellular dynamics, and activity of RBOHDs revealed that control of RBOH abundance and localization may play a fundamental role in the mechanism of ROS production in the context of plant defence responses.

## Materials and methods

### Materials

Tobacco BY-2 cells (*N. tabacum* cv. Bright Yellow-2) were grown at 25°C under continuous light (200 μE m^–2^ s^–1^) on a rotary shaker (140rpm). The suspensions were sub-cultured every 7 days at 2:80ml dilution, in MS medium (Murashige and Skoog, 1962) supplemented with 90mM sucrose, 1.5mM KH_2_PO_4_, 0.55mM inositol, 1 μM 2,4-D, 3 μM thiamine, and 10mM MES pH 5.6.

### ROS determination

Seven-day-old cells were harvested, filtered, and re-suspended (1g for 10ml) in I2 buffer (175mM mannitol, 0.5mM CaCl_2_, 0.5mM K_2_SO_4_, 2mM MES pH 5.8) for a 3h equilibration period on a rotary shaker (140rpm), then elicited by the addition of 50nM cryptogein. Cryptogein was purified from *P. cryptogea* according to Ricci *et al.* (1989). The production of H_2_O_2_ was measured by chemiluminescence using luminol and a luminometer (BCL book, Berthold). Every 2min, a 250 μl aliquot of the cell suspension was added to 50 μl of 0.3mM luminol and 300 μl of the assay buffer (175mM mannitol, 0.5mM CaCl_2_, 0.5mM K_2_SO_4_, 50mM MES pH 6.5).

### Plasmid constructions

The *RBOHD1* and *RBOHD2* cDNAs were obtained by reverse transcription of BY-2 total RNA using the ImProm-II™ reverse transcription system (Promega) and PCR amplification using primer pairs attB1-NoxD5/attB2-NoxD1-2 and attB1-NoxD5/attB2-NoxD2-1, respectively (primers are given in Supplementary Table S1 available at *JXB* online). The PCR products were cloned into Gateway entry vector pDONR221 (Invitrogen) for sequencing. The *RBOHD1* gene was PCR-amplified from BY-2 genomic DNA using primer pair attB1-NtrbohD4/attB2-NtrbohD1 and cloned into Gateway entry vector pDONR-Zeo (Invitrogen). It was then subcloned into plant transformation vector pMDC83 (Curtis and Grossniklaus, 2003) in which the *35S* promoter was deleted by digestion with restriction enzymes HindIII/SpeI, blunting of 5’-overhangs with Klenow fragment and re-circularization. The Golgi marker Man99-monomeric red fluorescent protein (mRFP) was a gift from Claude Saint-Jore-Dupas (Boulaflous *et al.*, 2009). It corresponds to the first 99 amino acids of *Glycine max* alpha-mannosidase I fused to mRFP.

### Plant cell transformation

The *RBOHD1-GFP* and *Man99-mRFP* constructs were introduced into *Agrobacterium tumefaciens* strain C58C1 by freeze thawing. *RBOHD1-GFP*-containing agrobacteria were used to transform BY-2 cells according to Brandizzi *et al.* (2003). Cells were plated onto agar-MS medium containing 35mg l^–1^ hygromycin. Transformed microcalli were transferred into MS liquid medium supplemented with 35mg l^–1^ hygromycin and submitted to constant agitation (140rpm) at 25°C under continuous light to generate cell suspensions. Eight weeks of subculturing were needed before cell suspension cultures became stable. *RBOHD1-GFP* cells were retransformed with *Man99-mRFP* construct and selected onto 100mg l^–1^ kanamycin to obtain the doubly transformed cell line.

### Real-time qRT-PCR analysis

Total RNA from 7-day-old BY-2 cells was isolated with the RNeasy Plant Mini Kit (Qiagen) and treated with Ambion DNA-*free*™ DNase (Life Technologies) to eliminate genomic DNA. First-strand cDNA was produced from 1 µg of total RNA using the ImProm-II™ reverse transcription system (Promega). The product was diluted 1:3 with water, and 2 µl was used as a template for RT-qPCR amplification with a GoTaq® qPCR Master Mix (Promega) on an ABI PRISM 7900HT system (Applied Biosystems). The housekeeping genes *EF-1α*, *L25*, and *PP2A*, whose expression has been shown to be stable in tobacco (Schmidt and Delaney, 2010), were used to normalize candidate gene transcripts. Primers were designed using Primer3 software (Untergasser *et al.*, 2012) and are listed in Supplementary Table S1 available at *JXB* online. Amplifications were carried out for three independent RNA preparations and two technical replications. Amplification specificity was checked by melting-curve analysis. PCR efficiency was determined using standard curves obtained with serial dilutions of PCR products as templates and shown to be close to 100% for all primer pairs. Data were analysed using the SDS 2.3 software (Applied Biosystems) to obtain cycle threshold values (Ct). Ct values were normalized to the geometric means of three reference genes (∆Ct) and relative expression values were calculated (2^–ΔCt^).

### Cell fractionation

Seven-day-old cells were collected by filtration, frozen in liquid nitrogen and homogenized in grinding medium (500mM sucrose, 20mM EDTA, 10mM DTT, 1mM PMSF, 50mM Tris-MES pH 8.0). The homogenate was centrifuged at 16 000*g* for 20min. After centrifugation, supernatants were collected, filtered through two successive screens (63 and 38 μM) and centrifuged at 96 000*g* for 35min. The microsomal fraction was purified by partitioning in an aqueous two-phase system containing polyethylene glycol 3350/dextran T-500, 6.6% each (Larsson *et al.*, 1994), to obtain the PM fraction, which was re-suspended in storage buffer (250mM sucrose, 1mM EDTA, 10mM DTT, 20% glycerol, 10 μg ml^–1^ leupeptin, 10 μg ml^–1^ pepstatin, 1mM phenylmethylsulfonyl fluoride, 10mM Tris-MES pH 7.3). The amount of protein present in the PM fraction was determined according to Bradford (1976) using bovine serum albumin (BSA) as standard.

### Western blot analysis

Samples of 20μg protein from PM fractions were solubilized in a buffer containing 40mM Tris-HCl pH 6.8, 5% β-mercaptoethanol, 1.5% SDS, 1mM EDTA, 2M/1M urea/thiourea, 1% *n*-octyl glucoside, 10% glycerol, and bromophenol blue for 2h at room temperature, then loaded on a 4–8% SDS-polyacrylamide gel. After electrophoresis separation (1h, 40 mA) protein fractions were electroblotted onto nitrocellulose membrane in a buffer containing 25mM Tris, 192mM glycine, 20% methanol, 0.1% SDS (2.5h, 200 mA). The membrane was blocked with TBS-Tween buffer (150mM NaCl, 0.05% Tween 20, 20mM Tris pH 7.6) containing 5% milk. Probing and detection of western blots were performed as described in the ECL Western Blotting detection kit (Amersham). The RBOHD antibody used for western blots and immunogold labelling (see below) was a rabbit polyclonal antibody raised against amino acids 138–152 (CLNKRPIPTGRFDRNK) and 784–798 (IAKNKGNKSGSASGGC) of RBOHD1 (Simon-Plas *et al.*, 2002). Dot-blot analysis revealed that the antibody only recognized oligopeptide 138–152. The fact that this oligopeptide is also present in the RBOHD2 sequence indicates that the antibody was able to detect both RBOHD isoforms. Primary anti-RBOHD and anti-GFP antibodies (Invitrogen) were used at a dilution of 1:1000 in TBS-Tween. A horseradish peroxidase anti-rabbit IgG antibody (Bio-Rad) was used at a 1:10 000 dilution in TBS-Tween for revelation.

### Confocal microscopy

Seven-day-old cells were mounted in culture medium or in I2 buffer when cryptogein treatment was required. FM4-64 (4.25 µM final) was added to the cell suspension in the dark at 25°C and labelling was imaged at different time points. Brefeldin A (BFA) (40 µM final) was added to the cell suspension 60min prior to imaging. Cryptogein (50nM final) and cycloheximide (CHX) (50 μg ml^–1^ final) were added alone or in combination, and effects were observed at different time points. Images were acquired using a Leica TCS SP2-AOBS laser scanning confocal microscope with the 488nm line of an argon laser for GFP and FM4-64, and with the 594nm line of a helium-neon laser for mRFP. GFP fluorescence was band-pass filtered between 500 and 550nm, FM4-64 fluorescence between 600 and 700nm, and mRFP fluorescence between 620 and 700nm. Images were processed using Photoshop CS5 (Adobe Systems). The PM fluorescence was quantified using image J software version 1.47h (Schneider *et al.*, 2012) as follows. One-pixel-wide lines (5–15 μm length) were drawn along the PM of at least 30 BY-2 cells per condition and time point, and average fluorescence intensity was measured. Fluorescence intensities were normalized to the time-0 point and graphed as percentages (mean ± SD; *n* = 5–16). Fluorescent intracellular compartments were scored at the periphery of the PM and scaled to 100 µm of PM using Image J. Compartment numbers were normalized to the time-0 point and graphed as percentages (mean ± SD; *n* = 6–11).

### High-pressure freezing and freeze substitution for transmission electron microscopy

The BY-2 cells suspended in I2 buffer were concentrated by centrifugation (2000*g*, 10 s), and immediately frozen, without addition of cryoprotectant, in a Leica EM HPM 100 high-pressure freezer. Freeze substitution was subsequently performed using automatic Leica EM AFS1 pre-cooled to –90°C. Samples were substituted in anhydrous acetone containing 0.2% uranyl acetate and 0.1% glutaraldehyde at **–**90**°**C for 72h. The temperature was gradually increased to **–**50**°**C (slope 5°C h^–1^) and kept at this temperature. Samples were rinsed with pure acetone, then pure ethanol for 24h for each solvent. They were then gradually infiltrated with mixtures of ethanol/Lowicryl HM20 (with increasing concentrations of resin) and finally embedded in pure Lowicryl HM20. Polymerization was carried out at –50°C for 48h, followed by 24h at –35°C, and finally 24h at 0°C. Following polymerization, the blocks of Lowicryl-embedded BY-2 cells were ready for thin sectioning and immunolabelling.

### Immunogold labelling of RBOHDs on thin sections of BY-2 cells and on purified PM

Ultrathin sections of tobacco cells were collected onto carbon-collodion-coated nickel grids. Grids were treated with 10mM PBS pH 7.2, supplemented with 10mM glycine for 15min, then with PBS containing 0.5% milk, 10% normal goat serum and Aurion blocking solution for 30min (Aurion), and then with polyclonal anti-RBOHD antibody at a 1:50 dilution in PBS containing 0.1% BSA-c (Aurion) for 1h at room temperature. Incubation with goat secondary antibody conjugated to 6nm gold particles (GPs; Sigma-Aldrich) and diluted 1:25 in PBS containing 0.1% BSA-c was then performed for 1h at room temperature. Purified BY-2 cell PM vesicles, pretreated or not with 20mM methyl-β-cyclodextrin (MβCD) in buffered conditions (150mM NaCl, 1mM PMSF, 20mM Tris pH 7.6) under constant agitation, were directly deposited onto collodion and carbon-coated microscope nickel grids. 10 µl of PM vesicles (0.2 µg µl^–1^) were deposited on each grid. Grids were floated, during 30min at room temperature, on 20 µl droplets of TBS containing 0.1% BSA, 0.1% glycine, and 5% normal goat serum (NGS), to reduce unspecific background. After three washing steps of 5min in droplets of TBS, the grids were incubated for 1h at RT with anti-RBOHD antibody or anti-GFP antibody diluted 1:50 in TBS. Antibody was detected with 5nm gold-labelled goat anti-rabbit IgG [EM GAR 5nm, British Biocell International (BBI)] or 5nm gold-labelled Protein A (EM protein A 5nm, BBI) at the dilution 1:20 for 45min at room temperature. After a 10min fixation in 0.1M phosphate buffer containing 2.5% glutaraldehyde, preparations were negatively stained during 30 s in 1% ammonium molybdate at room temperature and air-dried. Sections and grids were observed with a Hitachi H7500 transmission electron microscope operating at 80kV equipped with an AMT camera driven by AMT software (Hitachi).

### Labelling analysis and spatial statistics

In order to characterize PM vesicle labelling, the density of labelling was evaluated by counting the number of GPs per square micrometre of PM. When groups of GPs were identified on a vesicle, distances between all GPs in a group were measured with AMT software and the proportion of GPs in groups (2, 3, and 4 particles) was compared to total labelling. Counting and measurement were performed on three repetitions for each time of treatment and 30 PM vesicles per sample were observed. For spatial statistics, coordinates of the GPs and vesicle contours were determined using ImageJ software version 1.47h (Schneider *et al.*, 2012). The GP patterns within PM vesicles were considered as realizations of a stationary point process observed in windows of varying sizes and shapes. The spatial distribution of a stationary point process can be quantified based on Ripley’s K-function (Ripley, 1976). For a given radius *r*, K(r)=N(r)/λ where N(r) is the expected number of neighbours lying within distance *r* from a typical point and *λ* is the mean number of points per unit area. For a completely random (Poisson) point process, $K(r)=\pi r^{2}$ . When *K* is above $\pi r^{2}$, a point process is considered as spatial clustering. When *K* is below $\pi r^{2}$ , it is considered as spatially repulsive. Estimation of the K-function was performed using the translation correction (Ohser, 1983). Instead of computing individual estimates on each realization (PM vesicle), a global estimation was performed by pooling together all observed patterns using the approach proposed by Baddeley *et al.* (1993). Computation of the global estimates was implemented as a modified version of the Kest function provided by the R spatstat package (Baddeley *et al.*, 1993; R Developement Core Team, 2009). Simulation envelopes of the K-function under the Poisson hypothesis were computed from a modified version of the envelope function of spatstat.

## Results and discussion

### Two *RBOHD* genes are expressed in BY-2 cells

**Fig. 1.**
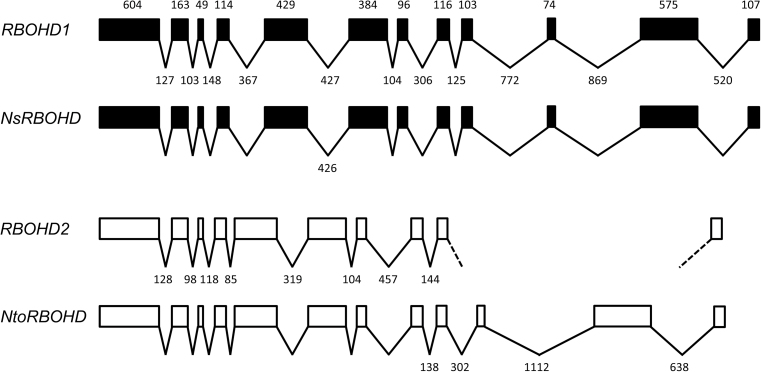
*RBOHD* gene structure in *N. tabacum* and parental species. Protein-coding regions are represented by boxes and introns by broken lines. The length of protein-coding regions in base pairs is marked above the *RBOHD1* sequence and is identical in all four genes. The length of introns is marked below the *RBOHD1* and *RBOHD2* genes and only marked below the *NsRBOHD* and *NtoRBOHD* genes when different from the tobacco genes. All sequences are drawn to scale. The *RBOHD1* gene sequence was completed after amplification and sequencing of BY-2 genomic DNA. A part of the *RBOHD2* gene near the 3’-end has not been found in the SGN database.

In order to express an RBOHD-GFP fusion protein at native level in tobacco BY-2 cells, a BLAST search of the SOL genomic network (SGN) database was performed with *RBOHD* cDNA (accession number AJ309006.1) to identify the tobacco *RBOHD* gene. The search identified two copies of the *RBOHD* gene, which were named *RBOHD1* and *RBOHD2* (Fig. 1). *N. tabacum* is a young allotetraploid species resulting from the hybridization of the diploid species *N. tomentosiformis* and *N. sylvestris* less than 200 000 years ago (Leitch *et al.*, 2008). A second BLAST search of the SGN database identified a single *RBOHD* gene in the *N. tomentosiformis* genome (*NtoRBOHD*) and *N. sylvestris* genome (*NsRBOHD*). The intron insertion pattern was identical in all four genes (Fig. 1). Intron length was different for *RBOHD1* and *RBOHD2*, but almost identical for *RBOHD1* and *NsRBOHD*, and for *RBOHD2* and *NtoRBOHD*. The cDNAs for *RBOHD1* and *RBOHD2* were successfully isolated from BY-2 cells. Both sequences shared 98.4% identity at the amino-acid level and 96.7% identity at the nucleotide level in their coding regions (Supplementary Figure S1 available at *JXB* online). Sequence comparison with *N. tomentosiformis* and *N. sylvestris RBOHD* coding regions showed that *RBOHD2* shared 100% identity at the nucleotide level with *NtoRBOHD* and *RBOHD1* displayed a single mismatch with *NsRBOHD* (data not shown). Altogether, the results indicate that *RBOHD1* and *RBOHD2* are two homeologous genes originating from *N. sylvestris* and *N. tomentosiformis,* respectively, that are transcribed in BY-2 cells.

### RBOHDs localize to the PM and endomembranes

**Fig. 2.**
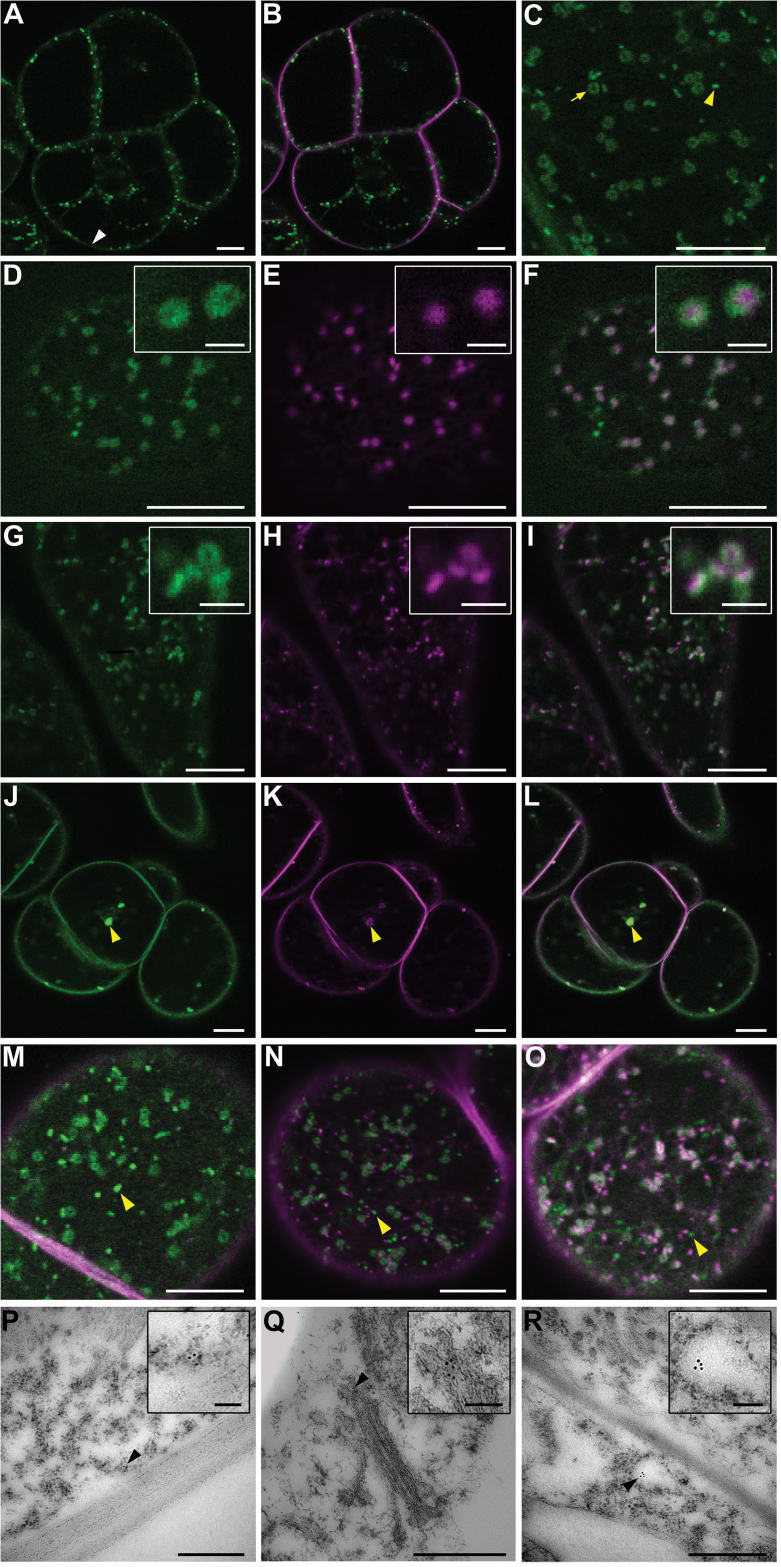
RBOHDs mostly localize to the PM and Golgi in BY-2 cells, as shown by confocal microscopy of RBOHD1-GFP-expressing BY-2 cells (A–O) and immunogold labelling of RBOHDs on ultrathin sections of wild-type cells (P–R). (A–C) Cells expressing RBOHD1-GFP and stained with the endocytic tracer FM4-64 for 5min. Shown are GFP fluorescence alone (A, C) and an overlay of GFP and FM4-64 fluorescence (B). GFP signals were observed at the PM [white arrowhead in (A)] and in intracellular dots and rings [yellow arrowhead and yellow arrow, respectively, in (C)]. (D–F) Cells co-expressing RBOHD1-GFP and the Golgi marker Man99-mRFP. Shown are GFP fluorescence (D), mRFP fluorescence (E), and overlay (F). The insets show that RBOHD1-GFP labelled the margin of the Golgi. (G–I) Cells expressing RBOHD1-GFP and stained with the endocytic tracer FM4-64 for 30min. Shown are GFP fluorescence (G), FM4-64 fluorescence (H), and overlay (I). The inset in (I) shows tricoloured labelling due to partial overlap. (J–L) Cells expressing RBOHD1-GFP, treated with BFA for 60min and with FM4-64 for 30min. Shown are GFP fluorescence (J), FM4-64 fluorescence (K), and overlay (L). The yellow arrowhead indicates a BFA body. (M–O) RBOHD1-GFP-expressing cells were stained with FM4-64 for 10min (M), 20min (N), and 60min (O). Shown are overlays of GFP and FM4-64 fluorescence. Yellow arrow and arrowheads indicate FM4-64-labelled tonoplast and green-only dots, respectively. (P–R) Immunogold-labelled sections of BY-2 cells performed with anti-RBOHD antibody. Black arrowheads indicate GPs associated with the PM (P), the margin of the Golgi (Q), and a vesicle-like compartment (R). Scale bar represents 10 µm (A–O), 2 µm (insets in panels D–I), 500nm (P–R), and 100nm (insets in panels P–R).

The *RBOHD1* gene including a **~**2-kb fragment upstream of the start codon was fused to GFP coding sequence and used to transform BY-2 cells. GFP fluorescence was detected at the PM and also intracellularly in the form of dots and rings (Fig. 2A–C). Similar fluorescence patterns were observed with *35S*-driven fusion constructs *RBOHD1-GFP*, *GFP-RBOHD1*, and *RBOHD2-GFP* suggesting that cell localization of the fusion protein is not significantly influenced by its expression level, the position of the fluorescent tag, or the identity of the isoform (data not shown).

Unlike their animal counterparts (Ushio-Fukai, 2009), plant NADPH oxidases have not been reported to be associated with intracellular compartments. This prompted us to identify the ones that are labelled by RBOHD1-GFP.

Fluorescent rings were previously observed in plant cells expressing Golgi fusion proteins (Robinson *et al.*, 2008). RBOHD1-GFP rings were identified as Golgi from the following results. Firstly, RBOHD1-GFP co-localized with the Golgi marker Man99-mRFP (Boulaflous *et al.*, 2009) and formed a rim around the Man99-GFP signal, suggesting a location at the periphery of the Golgi cisternae (Fig. 2D–F). Secondly, the RBOHD1-GFP signal was found in close apposition with that of FM4-64, an endocytic tracer that labels the *trans*-Golgi network (TGN) and the *trans* side of the Golgi stack 30min after internalization (Bolte *et al.*, 2004; Lam *et al.*, 2007) (Fig. 2G–I). Thirdly, RBOHD1-GFP labelling was sensitive to treatment with BFA, a fungal toxin that causes aggregation of Golgi stacks and TGN into BFA bodies (Ritzenthaler *et al.*, 2002). Upon BFA treatment, RBOHD1-GFP relocated to large aggregates that were also labelled by FM4-64 30 to 45min after uptake (Fig. 2J–L).

RBOHD1-GFP-labelled dots were not identified in the course of our study. Co-localization studies performed with FM4-64 over a 1h time course always showed the continuous presence of a population of GFP-only labelled dots (Fig. 2M–O). At time 60min, FM4-64 labelled the tonoplast (Fig. 2O). This result suggests that the unidentified compartment does not lie on the endocytic pathway and could correspond to an exocytic compartment that delivers RBOHDs from the Golgi to the PM via a TGN-independent route. A similar exit route from the Golgi complex has been suggested for the cellulose synthase complex in *Arabidopsis* (Crowell *et al.*, 2009).

Knowing that the location of a fusion protein may be different from that of its native form, ultrathin sections of BY-2 cells were immunogold-labelled with an antipeptide antibody that recognized the two RBOHD isoforms. Gold particles were observed at the PM, at the periphery of the Golgi cisternae, and associated with vesicle-like compartments (Fig. 2P–R), supporting the results of the live cell fluorescence microscopy studies. It should be mentioned that overall labelling density was low, either arguing for low abundance of the native RBOHDs or for poor epitope accessibility.

Altogether, the confocal and electron microscopy studies revealed that RBOHD isoforms were partitioned between the PM and endomembranes in BY-2 cells.

### RBOHDs are organized in clusters within the PM

**Fig. 3.**
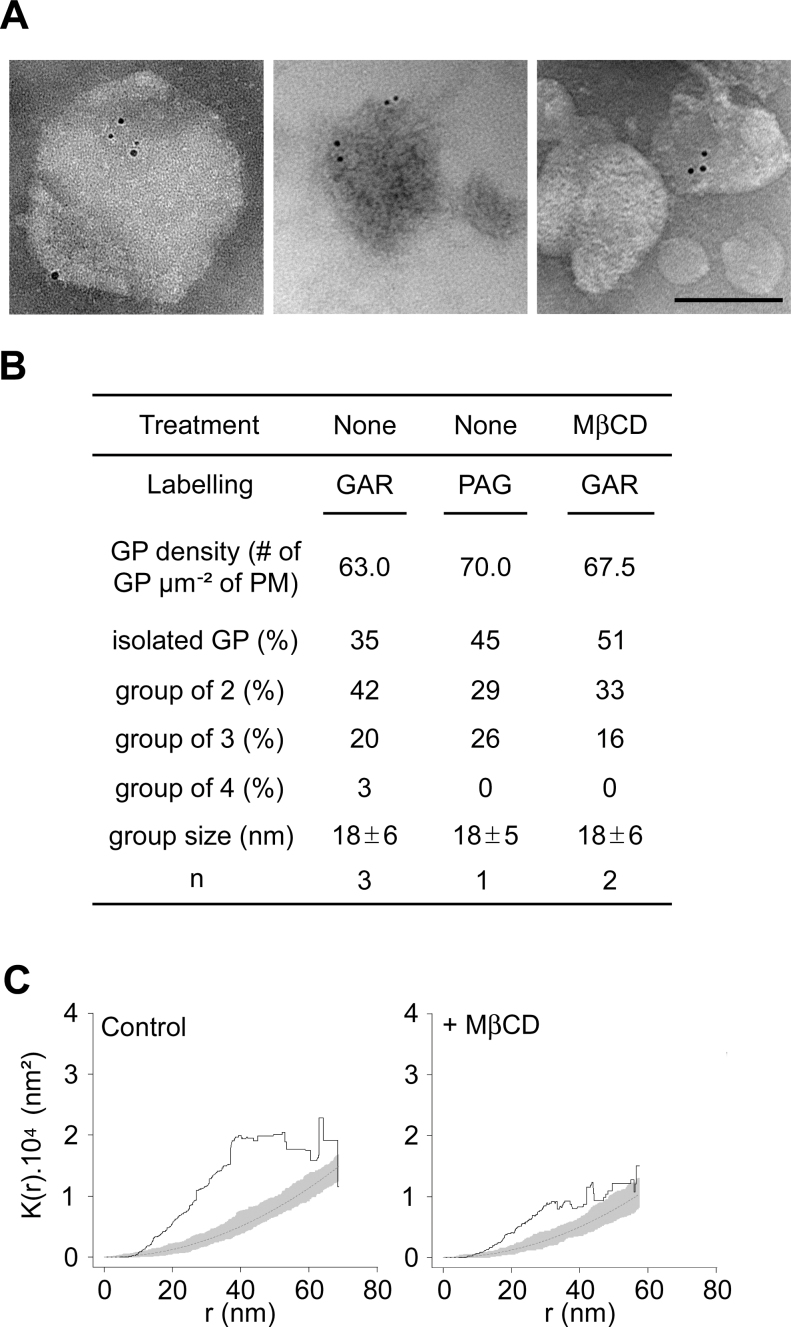
RBOHDs form clusters in the PM. (A) Representative transmission electron micrographs of BY-2 cell PM vesicle labelled with anti-RBOHD antibody and secondary IgG coupled to GPs of 5nm (GAR). Scale bar = 100nm. (B) Labelling characteristics of PM vesicles. Vesicles were treated or not with MβCD, then labelled with anti-RBOHD and either GAR or PAGs of 5nm (n, number of experiments). (C) Ripley’s K-function analysis of RBOHD distribution on PM vesicles. K(r) (y axis) is the average number of particles lying at a distance less than r (x axis) from a typical particle, normalized by the mean particle density. K(r) values displayed above simulation intervals for a completely random (Poisson) point pattern indicate an aggregated pattern. Black line, sample K(r); dotted line, theoretical Poisson K(r); grey area, 99% Poisson simulation interval.

Despite the low density of the labelling observed on BY-2 cell sections, GPs linked to the anti-RBOHD antibody mostly occurred as groups of 2–4 on cell PM and endomembranes (Fig. 2P–R). Purified PM vesicles of BY-2 cells were probed with the anti-RBOHD antibody to analyse the distribution pattern of RBOHDs on the PM surface (Fig. 3A). The mean density of the labelling over three independent biological experiments was 63 GPs µm^–2^ of PM (Fig. 3B). This value is low compared to the ones obtained on the same material for phosphatidylinositol 4,5-bisphosphate (PIP_2_) and H^+^-ATPase PMA2 (Furt *et al.*, 2010), which were, respectively, of 160 and 1200 µm^–2^ PM, but is in agreement with the low density of the labelling observed on cell sections. 65% of the labelling was observed as groups of GPs and 35% as isolated GPs (Fig. 3B). For aggregated labelling, the groups, with a mean size of 18±6nm, were 65% composed of two particles, 31% composed of three particles, and 4% composed of four particles. Statistical significance of the aggregated pattern of RBOHDs was evaluated with Ripley’s K function (Ripley, 1976), a spatial analysis method previously used to investigate the spatial distribution of animal membrane proteins (Prior *et al.*, 2003; Lillemeier *et al.*, 2006). K-function analysis confirmed that the gold pattern was aggregated, since K(r) values laid clearly above the simulation envelope for the K-function of completely random patterns (Fig. 3C, left panel). To rule out the possibility that groups might correspond to the binding of several secondary antibodies to a single primary antibody, a fourth experiment was performed with protein A-gold conjugates (PAG). The number of particles within groups was only slightly reduced (by 10%) in PAG-labelled vesicles compared to secondary antibody-labelled vesicles (Fig. 3B), clearly indicating that RBOHDs are organized in clusters at the PM. Labelling of PM vesicles prepared from RBOHD1-GFP cells with an anti-GFP antibody revealed that the fusion protein forms similar clusters in the PM (Supplementary Figure S3 available at *JXB* online), but with a lower density that could be due to the lower sensitivity of the GFP antibody and/or the lower abundance of the antigen.

Similarly, NADPH oxidase from human neutrophil was reported to be distributed in clusters within the PM (Wientjes *et al.*, 1997). In line with this finding are the non-uniform distribution of several plant RBOHs in the PM of differentiating cells (Takeda *et al.*, 2008; Liu *et al.*, 2009; Lee *et al.*, 2013) and the demonstration that H_2_O_2_ resulting from the activity of RBOHDs upon cryptogein treatment was observed as discrete patches along the PM of BY-2 cells (Lherminier *et al.*, 2009).

Such a clustering is also in agreement with previous experiments showing that RBOHDs are found in the DIM fractions of tobacco (Mongrand *et al.*, 2004; Morel *et al.*, 2006) or rice (Fujiwara *et al.*, 2009). Indeed, preferential association of a protein (or lipid) with sterol- and sphingolipid-enriched DIM fractions has been proven to be indicative of its presence in subdomains of the membrane in numerous studies (reviewed by Simons and Gerl, 2010). Like RBOHDs, the lipid PIP_2_, and the proteins remorin and flotillin, have been found predominantly associated with the DIM fractions of plant PM and detected by immuno-electron microscopy in PM nanodomains of 25, 70, and 100nm, respectively (Raffaele *et al.*, 2009; Furt *et al.*, 2010; Li *et al.*, 2012).

The sterol-chelating agent MβCD was used on PM vesicles to assess the possible sterol-dependency of the clustered distribution. A 30-min treatment with MβCD, which removed ~60% of PM sterols (data not shown), increased by 16% the number of isolated particles but did not modify either the labelling density or the size of the GP groups (Fig. 3B). Accordingly, K-function analysis of MβCD-treated vesicles indicated that the distribution of GPs was still clustered, although the amplitude of K curves was lower than for untreated vesicles, suggesting a decrease in the degree of aggregation after treatment with MβCD (Fig. 3C, right panel). This indicates that neither the association of RBOHD proteins to the PM nor their pattern within the membrane is fully dependent upon PM sterol content. Similar results have been obtained for several raft markers such as ganglioside M1 in mouse fibroblast PM (Fujita *et al.*, 2007), a tyrosine kinase lck-derived oligopeptide in mouse T-cell PM (Lillemeier *et al.*, 2006), and PIP_2_ in tobacco cell PM (Furt *et al.*, 2010).

The small and homogeneous size of the RBOHDs clusters, together with their relative resistance to cyclodextrin, suggests that RBOHDs might be present in plant membranes as oligomers. Interestingly, Nox5, an animal NADPH oxidase which is structurally more closely related to plant RBOHs than any of the other animal isoforms (Suzuki *et al.*, 2011), forms an active oligomer in the PM (Kawahara *et al.*, 2011). Moreover, structure and physiological studies of rice RBOHB revealed that it could form a functional dimer (Oda *et al.*, 2010). This does not question the fact that RBOHD clusters might reside in particular sterol-rich domains of the membrane. Indeed, in animal cells, several classes of proteins have been demonstrated to be associated with membrane domains as oligomers. For instance, the matrix protein VP40 from the Ebola virus is found essentially in the DIM fractions as oligomers whereas the low amount of the protein present in the soluble fraction consists mostly of monomers (Panchal *et al.*, 2003). For members of the flotillin family, found associated to membrane rafts in various models and proven to form oligomers, oligomerization is necessary to mediate association with DIMs (Neumann-Giesen *et al.*, 2004). Furthermore, oligomerization of the amyloid β-peptide is ganglioside-dependent and MβCD-insensitive within lipid rafts of CHO cells (Kim *et al.*, 2006). Finally, a very interesting study with glycosylphosphatidylinositol-anchored proteins in MDCK epithelial cells identified oligomerization as one of the determinants of their association to sterol-rich domains in the Golgi and their polarized transport to the apical cell membrane (Paladino *et al.*, 2004).

All these data are thus consistent with the hypothesis that RBOHDs might reside as oligomers within sterol-rich domains of the tobacco PM. Further investigations such as blue native gel and radiation inactivation analyses will be required to ascertain the oligomeric nature of RBOHDs.

### Intracellular and PM-associated pools of RBOHDs are differentially affected following cryptogein treatment

Elicitation of tobacco cells by cryptogein induces a rapid and transient production of ROS (Viard *et al.*, 1994) that depends upon the activity of RBOHDs (Simon-Plas *et al.*, 2002). In BY-2 and RBOHD1-GFP-expressing cells, ROS production peaked 10–15min after cryptogein addition then decreased to a level slightly above basal levels at 30min (Supplementary Figure S2 available at *JXB* online). ROS production was consistently higher in RBOHD1-GFP-expressing cells suggesting that the fusion protein retained some enzymatic activity as previously reported for an N-terminal fusion (Takeda *et al.*, 2008). As this is not direct evidence for activity, one has to keep in mind that the fusion enzyme could exhibit a reduced enzymatic activity due to the presence of the fluorescent tag. To better understand the transient production of ROS, the subcellular distribution of RBOHDs was investigated within the first hour of elicitation.

**Fig. 4.**
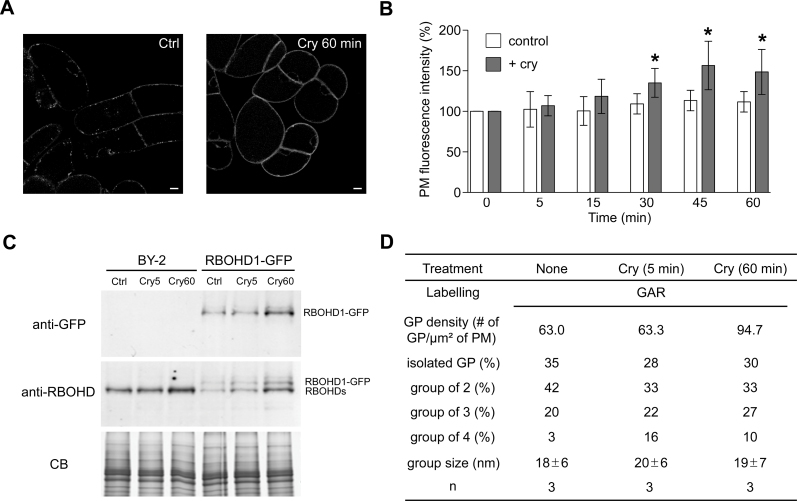
PM abundance of RBOHDs increases upon elicitation by cryptogein. (A) RBOHD1-GFP-expressing cell before (Ctrl) and after 60min incubation with cryptogein (Cry 60min). Scale bars = 10 µm. (B) Quantification of the PM fluorescence in RBOHD1-GFP cells. Values are means ± SD of 5–16 independent experiments. An asterisk indicates a difference of statistical significance between non-elicited and elicited cells for each time point, Mann-Whitney test (*P* < 0.05). (C) Western blot analysis of PM fractions from BY-2 and RBOHD1-GFP cells with anti-RBOHD and anti-GFP antibodies. Cells were untreated (Ctrl) or cryptogein-treated for 5min (Cry5) or 60min (Cry60). CB: Coomassie blue staining as a loading control. (D) RBOHD distribution on PM vesicles from elicited BY-2 cells. Values for non-elicited cells are the same as the ones reported in Fig. 2B. Abbreviation: n, number of experiments.

The first obvious change observed in elicited RBOHD1-GFP cells was an increase in PM fluorescence (Fig. 4A). PM fluorescence intensity of RBOHD1-GFP cells was significantly different from that of untreated cells at 30min and was ~40% higher at 60min (Fig. 4B). PM accumulation of the fusion protein was also shown by western blot analysis of PM-enriched fractions using GFP antibody (Fig. 4C). Furthermore, immunodetection with an antibody recognizing both RBOHD1 and RBOHD2 revealed that native isoforms accumulate at the PM 60min after elicitation (Fig. 4C). Immunolabelling of PM vesicles also showed a 50% increase in the number of GPs (Fig. 4D). All these approaches thus yielded a similar result, namely an increase of about 50% of the amount of RBOHDs present on the PM after 1h of cryptogein treatment, and further confirmed that the fusion protein is a faithful reporter for monitoring RBOHDs subcellular dynamics.

**Fig. 5.**
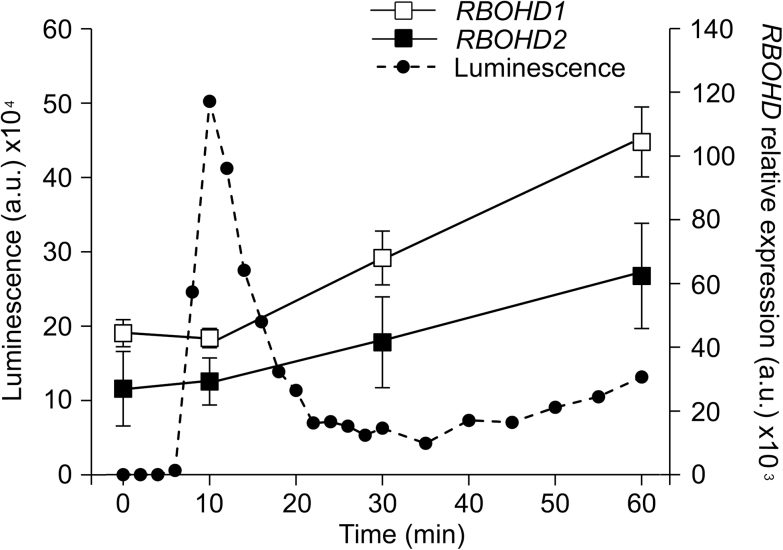
Kinetics of ROS production and *RBOHD* gene expression following elicitation. The kinetics of ROS production, measured by chemiluminescence (dashed line, left axis), is plotted against the kinetics of *RBOHD1* and *RBOHD2* mRNA accumulation, measured by qRT-PCR (solid lines, right axis). ROS kinetics is representative of three independent experiments. Expression values are means ± SEM of log2-transformed mRNA levels relative to the geometric mean of reference genes *EF-1α*, *L25*, and *PP2A* (*n* = 3). Values are expressed in arbitrary units (a.u.).

**Fig. 6.**
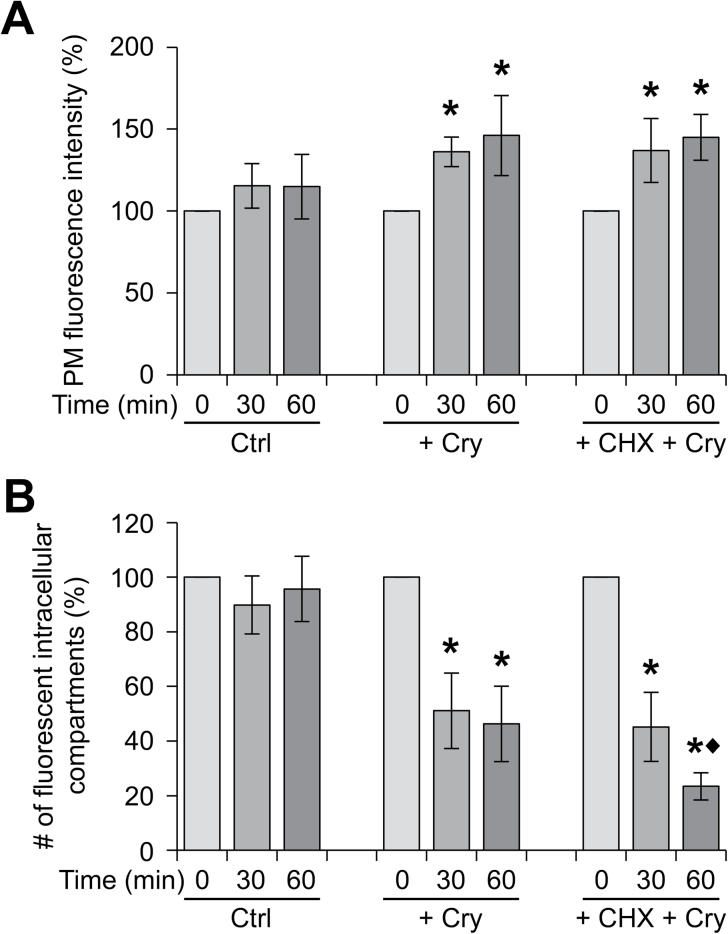
RBOHDs are mobilized from the internal compartments to the PM upon elicitation by cryptogein. RBOHD1-expressing cells were untreated (Ctrl), or treated with cryptogein alone (Cry), or in combination with the protein synthesis inhibitor CHX. PM fluorescence (A) and fluorescent intracellular compartments (B) were quantified for 10–30 cells for each time point. Values are means ± SD of 6–11 independent experiments. A difference of statistical significance is indicated by an asterisk (control vs treated cells) or a black diamond (Cry-treated vs Cry + CHX-treated cells) for each time point. Mann-Whitney test, *P* < 0.05.

Simon-Plas and collaborators previously showed that cryptogein upregulates *RBOHD* transcription in tobacco cells (Simon-Plas *et al.*, 2002). This was confirmed in the present study using real-time PCR. *RBOHD1* and *RBOHD2* transcript levels were upregulated ~1.5-fold 30min after elicitation and ~2.3-fold at time 60min (Fig. 5). Transcriptional upregulation could explain the 50% increase in abundance of RBOHDs at the PM. However, treatment of RBOHD1-GFP-expressing cells with the protein synthesis inhibitor CHX had very little effect on the cryptogein-triggered increase in PM fluorescence (Fig. 6A). This indicates that RBOHD1-GFP accumulation at the PM after exposure to cryptogein is not mainly due to the delivery of newly synthesized proteins.

At the same time as the increase in PM fluorescence, a decrease in the number of fluorescent intracellular compartments was observed in elicited cells. Indeed, cryptogein induced a 2-fold reduction in the number of these compartments 30min after treatment (Figs 4A and 6B). A mechanistically relevant explanation is that cryptogein triggers the targeting to the PM of RBOHDs released from intracellular stores. The combination of cryptogein and CHX led to a decrease in the number of fluorescent intracellular compartments 60min after elicitation which was much more pronounced than with cryptogein alone, suggesting that internal stores are both the early source of RBOHD proteins accumulating at the PM and the late destination of newly synthesized proteins arriving from the endoplasmic reticulum.

Altogether these results are in favour of a distribution of RBOHDs between PM and Golgi pools that is shifted in favour of the PM pool following elicitation, first by the delivery of proteins already present in the Golgi, and then by the synthesis of new proteins. This raises the question of the Golgi as a genuine reservoir for RBOHDs. There is no report in the literature of any Golgi-localized NADPH oxidase in plant or animal cells. However, there are several examples of PM-associated proteins that cycle between the PM and the Golgi (Nichols *et al.*, 2001; Le and Nabi, 2003; Milhas *et al.*, 2010). Interestingly, these proteins have been found associated with lipid rafts. Raft assembly is presumed to be initiated at the ER with proteins being incorporated at the Golgi complex (Brown and London, 1998; Heino *et al.*, 2000). Nichols and collaborators have raised the possibility that recycling of PM raft components could play a role in raft assembly and in the regulation of signalling pathways that are lipid raft-dependent (Nichols *et al.*, 2001). Further experiments involving pharmacological and/or genetic alterations of the endocytic and exocytic pathways, coupled with the use of photoswitchable fluorescent proteins, will be required to demonstrate that a similar cycling operates in plant cells to regulate the abundance of RBOHD at the PM.

### Insights on the regulation of RBOHD activity

Since RBOHD-mediated ROS production was observed a few minutes after elicitation with cryptogein (Fig. 5), we asked whether RBOHD activation was associated with their redistribution in the PM. The percentage of GPs within groups and the size of the groups did not change significantly 5min after elicitation (Fig. 4D), indicating that activation of the protein that occurs within this time frame does not result from the aggregation of enzyme-containing nanodomains to generate large redox platforms as documented for animal cells (Jin *et al.*, 2011). Our results, however, do not rule out the possibility that signalling platforms are formed from the coalescence of nanodomains containing different sets of signalling components including RBOHDs, or targeting of regulatory proteins to RBOHD nanodomains. Consistent with the latter hypothesis is the observation that DIMs extracted from BY-2 cells, treated or not with cryptogein for 5min, displayed the same amount of RBOHD, whereas the abundance of proteins involved in signal transduction, such as 14-3-3 proteins, was increased (Stanislas *et al.*, 2009). At time 60min, when new RBOHDs accumulated at the PM, the percentage of isolated GPs as well as the size of the groups also remained unchanged (Fig. 4D), suggesting that RBOHDs reach the PM as clusters that might have been assembled at the rim of the Golgi cisternae (Fig. 2Q).

Another interesting aspect of RBOHD regulation is the discrepancy between the kinetics of *RBOHD* transcript accumulation and ROS production upon cryptogein treatment. Indeed, *RBOHD1* and *RBOHD2* transcript levels were unaffected 10min after elicitation (Fig. 5) indicating that ROS production results from the activation of a PM-resident pool of enzymes. Moreover, the fact that arrival of new RBOHDs at the PM coincides with the decrease in ROS production is fully consistent with previous results indicating that PM-localized ROS production is no longer detected after 30min of elicitation (Lherminier *et al.*, 2009). This suggests that activated enzymes are turned over and replaced by inactivated ones, thus enabling the PM rapidly to restore its signalling capacity and giving time for the cell to synthesize new enzymes. The catalytic core of the phagocytic NADPH oxidase cycles between the PM and internal reservoirs in macrophages, and it has been proposed that cycling could represent a mechanism by which superoxide production is regulated (Casbon *et al.*, 2009; Ejlerskov *et al.*, 2012).

## Conclusion

The results presented here allow a new hypothesis concerning the regulation of plant NADPH oxidases during the set-up of plant defence. Until now, this regulation has mainly been addressed by considering variation of gene expression, post-translational modifications, or regulation by other proteins or secondary messengers such as calcium or phosphatidic acid. It now seems of interest to consider subcellular trafficking as a potential determinant of RBOH activity. Future prospects will be to decipher the mechanisms underlying the subcellular dynamics of RBOHs and to evaluate the roles of the different pools of RBOHs in the signalling process associated with plant defence.

## Supplementary material

Supplementary data can be found at *JXB* online.

Supplementary Table S1. Primers used in this study.

Supplementary Figure S1. Alignment of RBOHD1 and RBOHD2 amino acid sequences.

Supplementary Figure S2. Kinetics of ROS production upon elicitation by cryptogein.

Supplementary Figure S3. RBOHD-GFP protein forms clusters in the PM.

## Funding

This work was supported by grants from the French Ministère de l’Enseignement Supérieur et de la Recherche; Institut National de la Recherche Agronomique (INRA); and the Grant Agency of the Czech Republic (P501/12/0590 to P.M.).

## Supplementary Material

Supplementary Data

## Abbreviations:

BFA: brefeldin A
BY-2: Bright Yellow-2
CHX: cycloheximide
DIM: detergent-insoluble fraction
GAR: goat anti-rabbit IgG
GP: gold particle
HR: hypersensitive response
MβCD: methyl-β-cyclodextrin
mRFP: monomeric red fluorescent protein
PAG: protein A–gold conjugate
PIP_2_: phosphatidylinositol 4,5-bisphosphate
PM: plasma membrane
RBOH: respiratory burst oxidase homologue
ROS: reactive oxygen species
SAR: systemic acquired resistance
TGN: *trans*-Golgi network.
