# Supplementary material for: Dynamic changes in the subcellular distribution of the tobacco ROS-producing enzyme RBOHD in response to the oomycete elicitor cryptogein
Source: J Exp Bot. 2014 Jul 1;65(17):5011–22. doi: 10.1093/jxb/eru265 (PMC4144778; doi:10.1093/jxb/eru265)
Supplement: Supplementary Data [file supp_65_17_5011__index.html]

Dynamic changes in the subcellular distribution of the tobacco ROS-producing enzyme RBOHD in response to the oomycete elicitor cryptogein — Dynamic changes in the subcellular distribution of the tobacco ROS-producing enzyme RBOHD in response to the oomycete elicitor cryptogein — Supplementary Data 

# Dynamic changes in the subcellular distribution of the tobacco ROS-producing enzyme RBOHD in response to the oomycete elicitor cryptogein

## Supplementary Data

Data files

**Files in this Data Supplement:**

- Supplementary Data - Supplementary Data
