# Supplementary material for: Dynamic changes in the subcellular distribution of the tobacco ROS-producing enzyme RBOHD in response to the oomycete elicitor cryptogein
Source: J Exp Bot. 2014 Jul 1;65(17):5011–22. doi: 10.1093/jxb/eru265 (PMC4144778; doi:10.1093/jxb/eru265)
Supplement: Supplementary Data [file supp_eru265_jexbot122317_file001.pdf]

Journal of Experimental Botany

Subcellular localization and dynamics of the tobacco ROS-producing enzyme RBOHD upon cryptogein elicitation

Elodie Noirot, Christophe Der, Jeannine Lherminier, Franck Robert, Pavla Moricova, Kiên Kiêu, Nathalie Leborgne-Castel, Françoise Simon-Plas, and Karim Bouhidel

```
RBOHD1        MQNSENHHPHHQHHHSDTEIIGNDRASYSGPLSGPLNKRGGKKSARFNIPESTDIGTSVG 60
RBOHD2        MQNSENHHPHHHHHHSDTEIIGNDRASYSGPLSGPLNKRGGKKSARFNIPESTDIGTSVG 60
              ***********:************************************************

RBOHD1        TGGKSNDDAYVEITLDVREDSVAVHSVKTAGGDDVEDPELALLAKGLEKKSTLGSSLVRN 120
RBOHD2        TGAKSNDDAYVEITLDVREDSVAVHSVKTAGGDDVEDPELALLAKGLEKKSTLGSSLVRN 120
              **.*********************************************************

RBOHD1        ASSRIRQVSQELRRLASLNKRPIPTGRFDRNKSAAAHALKGLKFISKTDGGAGWAAVEKR 180
RBOHD2        ASSRIRQVSQELRRLASLNKRPIPTGRFDRNKSAAAHALKGLKFISKTDGGAGWAAVEKR 180
              ************************************************************

RBOHD1        FDEITASTTGLLPRAKFGECIGMNKESKEFAVELYDALARRRNITTDSINKAQLKEFWDQ 240
RBOHD2        FDEITASTTGLLPRAKFGECIGMNKESKEFAVELYDALARRRNITTDSINKAQLKEFWDQ 240
              ************************************************************

RBOHD1        VADQSFDSRLQTFFDMVDKDADGRITEEEVREIIGLSASANRLSTIQKQADEYAAMIMEE 300
RBOHD2        VADQSFDSRLQTFFDMVDKDADGRITEEEVREIIGLSASANRLSTIQKQADEYAAMIMEE 300
              ************************************************************

RBOHD1        LDPNNLGYIMIENLEMLLLQAPNQSVQRGGESRNLSQMLSQKLKHTQERNPIVRWYKSFM 360
RBOHD2        LDPNNLGYIMIENLEMLLLQAPNQSVQRGGESRNLSQMLSQKLKHTQERNPIVRWYKSFM 360
              ************************************************************

RBOHD1        YFLLDNWQRVWVLLLWIGIMAGLFTWKYIQYKEKAAYKVMGPCVCFAKGAAETLKLNMAI 420
RBOHD2        YFLLDNWQRVWVLLLWIGIMAGLFTWKYIQYKEKAAYKVMGPCVCFAKGAAETLKLNMAI 420
              ************************************************************

RBOHD1        ILFPVCRNTITWLRNKTRLGAAVPFDDNLNFHKVIAVAIALGVGIHGLSHLTCDFPRLLN 480
RBOHD2        ILLPVCRNTITWLRSKTRLGAAVPFDDNLNFHKVIAVAIALGVGVHGLSHLTCDFPRLLN 480
              **:***********.*****************************:***************

RBOHD1        ASEEEYEPMKYYFGDQPESYWWFIKGVEGVTGIIMVVLMAIAFTLATPWFRRNRVSLPKP 540
RBOHD2        ASEEEYEPMKYYFGDQPESYWWFIKGVEGVTGIIMVVLMAIAFTLATPWFRRNRVSLPKP 540
              ************************************************************

RBOHD1        FHKLTGFNAFWYSHHLFVIVYTLFIVHGEKLYITKDWYKRTTWMYLTIPIILYASERLIR 600
RBOHD2        FHKLTGFNAFWYSHHLFVIVYTLFIVHGEKLYITKDWYKRTTWMYLTIPIILYASERLIR 600
              ************************************************************

RBOHD1        AFRSSIKAVKILKVAVYPGNVLALHMSKPQGYKYKSGQYMFVNCAAVSPFEWHPFSITSA 660
RBOHD2        ALRSSIKAVKILKVAVYPGNVLALHMSKPQGYKYKSGQYMFVNCAAVSPFEWHPFSITSA 660
              *:**********************************************************

RBOHD1        PGDDYLSVHIRTLGDWTRQLKTVFSEVCQPPPNGKSGLLRADYLQGENNPNFPRVLIDGP 720
RBOHD2        PGDDYLSVHIRTLGDWTRQLKTVFSEVCQPPPNGKSGLLRADYLQGENNPNFPRVLIDGP 720
              ************************************************************

RBOHD1        YGAPAQDYKKYEVVLLVGLGIGATPMISIVKDIVNNMKAMDEEENSLEDGHNNNMAPNSS 780
RBOHD2        YGAPAQDYKKYEVVLLVGLGIGATPMISIVKDIVNNMKAMDEEENSLENGDNNNMAQNSS 780
              ************************************************:*.***** ***

RBOHD1        PNIAKNKGNKSGSASGGNNFNTRRAYFYWVTREQGSFDWFKGIMNEAAEMDHKGVIEMHN 840
RBOHD2        PNIAQKRGNKSDSASGRNNFNTRRAYFYWVTREQGSFDWFKGIMNEAAEMDHKGVIEMHN 840
              ****:::****.**** *******************************************

RBOHD1        YCTSVYEEGDARSALITMLQSLHHAKNGVDIVSGTRVKSHFAKPNWRNVYKRIALNHPEA 900
RBOHD2        YCTSVYEEGDARSALITMLQSLHHAKNGVDIVSGTRVKSHFAKPNWRNVYKRIALNHPDA 900
              **********************************************************:*

RBOHD1        KVGVFYCGAPALTKELRQHALDFSHKTSTKFDFHKENF 938
RBOHD2        KVGVFYCGAPALTKELRQHALDFSHKTSTKFDFHKENF 938
              **************************************
```

Supplementary Fig. S1. Alignment of RBOHD1 and RBOHD2 amino acid sequences. Alignment was realized using ClustalW (Larkin et al., 2007). Fourteen amino acid differences are found in the two tobacco genes.

Larkin MA, Blackshields G, Brown NP, Chenna R, McGettigan PA, McWilliam H, Valentin F, Wallace IM, Wilm A, Lopez R, et al. 2007. Clustal W and Clustal X version 2.0. *Bioinformatics*. **23,** 2947-2948.

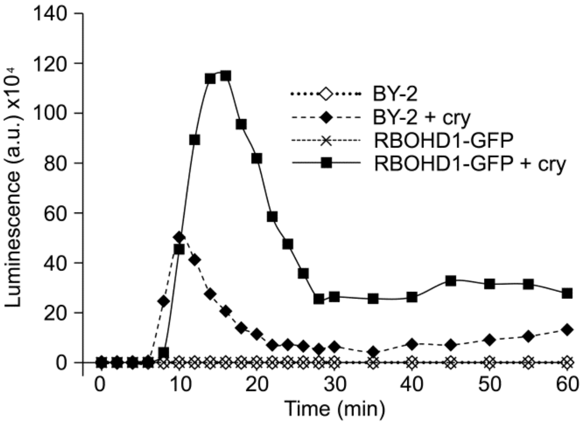

Supplementary Fig. S2. Kinetics of ROS production upon elicitation by cryptogein. ROS burst was measured in wild-type and NtrbohD1-expressing BY-2 cells by chemiluminescence (a.u.: arbitrary units). Kinetics is representative of 3 independent experiments.

A

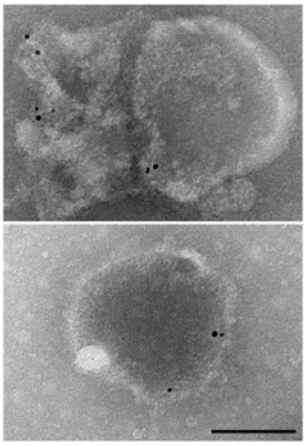

B

Treatment	None
Labelling	GAR
GP density (# of GP µm⁻² of PM)	27
isolated GP (%)	37
group of 2 (%)	32
group of 3 (%)	24
group of 4 (%)	7
group size (nm)	19±7
n	2

C

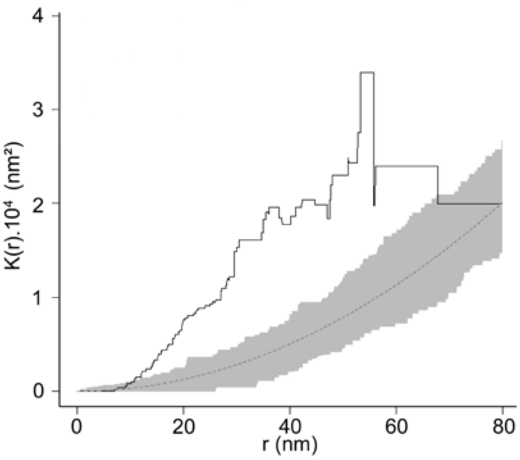

Supplementary Fig. S3. RBOHD-GFP protein forms clusters in the plasma membrane. (A) Representative transmission electron micrographs of PM vesicles from RBOHD-GFP-expressing cells and labelled with anti-GFP antibody and secondary IgG coupled to GPs of 5 nm (GAR). (Scale bar = 100 nm). (B) Labelling characteristics of PM vesicles. n = number of experiments. (C) Ripley's K-function analysis of RBOHD-GFP distribution on PM vesicles. Black line: sample K(r), dotted line: theoretical Poisson K(r), grey area: 99% Poisson simulation interval.

Supplementary Table S1. Primers used in this study

Cloning primers

**Primer name**	**Sequence**
attB1-NoxD5	5'-GGGGACAAGTTTGTACAAAAAAGCAGGCTAATGCAAAATTCGGAAAAT
attB2-NoxD1-2	5'-GGGGACCACTTTGTACAAGAAAGCTGGGTCAACCTGTGTCCTTAGCTG
attB2-NoxD2-1	5'-GGGGACCACTTTGTACAAGAAAGCTGGGTATAGGGAGAGGTGGTAGATTC
attB1-NtrbohD4	5'-GGGGACAAGTTTGTACAAAAAAGCAGGCTTCGTTTATTTAGGGCAAGTT
attB2-NtrbohD1	5'-GGGGACCACTTTGTACAAGAAAGCTGGGTAGAAATTTTCTTTATGGAAATCAAAC

qRT-PCR primers

**Gene name**	**Sequence**	
EF-1a	Forward	5'-TGAGATGCACCACGAAGCTC
	Reverse	5'-CCAACATTGTCACCAGGAAGTG
L25	Forward	5'-CCCCTCACCACAGAGTCTGC
	Reverse	5'-AAGGGTGTTGTTGTCCTCAATCTT
PP2A	Forward	5'-GTGAAGCTGTAGGGCCTGAGC
	Reverse	5'-CATAGGCAGGCACCAAATCC
RBOHD1	Forward	5'-CATCAAAACAGCTAAGGACACAG
	Reverse	5'-GTACACAATAGGGAGAGTTGGTAGAC
RBOHD2	Forward	5'-AGATACCAAGGGAATTAAGAATGTG
	Reverse	5'-GGCACCCATCAAAAGAGG
